# Engineering Na−Mo−O/Graphene Oxide Composites with Enhanced Electrochemical Performance for Lithium Ion Batteries

**DOI:** 10.1002/open.201900205

**Published:** 2019-08-29

**Authors:** Jingfa Li, Qiang Chen, Qihao Zhou, Nan Shen, Min Li, Cong Guo, Lei Zhang

**Affiliations:** ^1^ School of Chemistry and Materials Science Nanjing University of Information Science and Technology, Nanjing Jiangsu 210044 China; ^2^ School of Physics and Optoelectronic Engineering Nanjing University of Information Science and Technology, Nanjing Jiangsu 210044 China; ^3^ School of Atmospheric Physics Nanjing University of Information Science and Technology, Nanjing Jiangsu 210044 China

**Keywords:** sodium molybdate, graphene oxide, ultra-long nanorods, lithium ion batteries, energy storage

## Abstract

Sodium molybdate (Na−Mo−O) wrapped by graphene oxide (GO) composites have been prepared via a simple in‐situ precipitation method at room temperature. The composites are mainly constructed with one dimension (1D) ultra‐long sodium molybdate nanorods, which are wrapped by the flexible GO. The introduction of GO is expected to not merely provide more active sites for lithium‐ions storage, but also improve the charge transfer rate of the electrode. The testing electrochemical performances corroborated the standpoint: The Na−Mo−O/GO composites delivers specific capacities of 718 mAh g^−1^ after 100 cycles at 100 mA g^−1^, and 570 mAh g^−1^ after 500 cycles at a high rate of 500 mA g^−1^; for comparison, the bare Na−Mo−O nanorod shows a severe capacity decay, which deliver only 332 mAh g^−1^ after 100 cycles at 100 mA g^−1^. In view of the cost‐efficient and less time‐consuming in synthesis, and one‐step preparation without further treatment, these Na−Mo−O nanorods/GO composites present potential and prospective anodes for LIBs.

Nowadays, lithium ion batteries (LIB) are considered as the dominant energy sources of choice for electronic vehicles and mobile devices because of their high volumetric and gravimetric capacity.^1^ Currently graphite is the commercial anode counterpart, however, it comes with the disadvantage of limited theoretical gravimetric capacity. (LiC_6_ stoichiometry ∼372 mAh g^−1^).^2^ Therefore there is a strong motivation to exploit high‐capacity alternatives to graphite. Inorganic molybdates attract particular interest since a high capacity can be delivered due to the reduction of Mo^6+^ to Mo^0^ with the 6 electron change transferring. Besides, the ease of reduction of Mo^6+^ causes conversion type reactions to happen at lower voltages vs. Li^+^/Li, thereby qualifying the Mo^6+^ containing oxides as potentially suitable anode materials for LIBs. Sodium containing Mo oxides such as Na_0.23_MoO_3_,^3^ Na_2_MoO_4_,^4^ Na_2_Mo_2_O_7_,^5^ due to its interesting structure and thermodynamic stability,^6^ have been explored as possible anode materials for LIBs. However, the electronic conductivities of these materials are not satisfactory in most cases. Thus, various strategies have been explored to improve the electrochemical behaviors. Among them, decreasing the particle size or at least certain dimension to nanoscale is an effective way to shorten the ions diffusion path.^7^

One dimension (1D) nanostructure, such as nanowires, nanotubes, nanofibers and nanorods, are always the focuses of researchers for the application of energy conversion and storage.^8^, ^9^, ^10^, ^11^, ^12^ That is because 1D nanomaterials could provide large surface‐to‐volume ratio, shortened ion/charge diffusion distance and facile strain accommodation upon lithiation and delithiation processes for LIBs, thus resulting in improved electrochemical performances. The past decade had witnessed significant progress in the synthesis of 1D nanostructured materials, such as electrospinning fiber technique,^8^ hydrothermal method and calcination,^13^ self‐sacrificial template method,^14^ most of which however require much cost and complicated procedure. Apart from the nanotechnologies, coupling with carbonaceous materials is another effect way to enhance the electrochemical performance by improving the electronic conductivity of the electrodes.^15^

Herein, we proposed a very facile route to obtain the Na−Mo−O nanorods with the aspect ratio of 700 at room temperature. Moreover, the Na−Mo−O nanorods/GO composites were also fabricated to improve the electronic conductivity and structure integrity, in which the ultralong 1D Mo−O nanorods were in‐situ precipited upon the surface of flexible GO. As expected, the Na−Mo−O nanorods/GO composites as the anode could realize the enhanced electrochemical performance: it delivers a capacity of 718 mAh g^−1^ after 100 cycles at 100 mA g^−1^, and 570 mAh g^−1^ after 500 cycles at a high rate of 500 mA g^−1^; both of the values are much higher than that of bare Na−Mo−O nanorods. Given the enhanced ability and the low cost efficiency compared with the reduced GO, (the transformation from GO into rGO need the reduction process through the chemical reduction by hydrazine hydrate (N_2_H_4_ ⋅ H_2_O) or thermal reduction), the Na−Mo−O nanorods/GO composites shows great potential in the practical applications of LIB anodes.

The simple engineering process of Na−Mo−O nanorods and Na−Mo−O nanorods/GO composites is illustrated in Figure 1a. As schematic in Step I, a rather facile step involves the precipitant reaction at room temperature to produce the Na−Mo−O nanorods. In step II, GO were firstly dispersed in the water solvent to form a homogeneous solution feasibly before adding the reactants. After that, the Na−Mo−O nanorods were growing along the single layer orientation of graphene oxides and finally Na−Mo−O nanorods/GO composites were formed with compounding nanorods with thin graphene layer. X‐ray diffraction (XRD) was firstly adopted to characterize the phase structures of the as‐obtained samples. As shown in Figure 1b, the XRD pattern can mainly be indexed to Na_2_Mo_2_O_7_ (JCPDF card No. 01‐076‐0185) with the impurity of Na_2_MoO_4_ (JCPDF card No. 00‐026‐0968). No more obvious difference between the Na−Mo−O nanorods and Na−Mo−O nanorods/GO composites was observed through the XRD patterns, indicating the integrity of the Na−Mo−O nanorods and graphene oxides component. Energy dispersive X‐ray spectrum (EDX) in Figure 1c was adopted to semi‐quantitatively collect the information of the element ratio: The Na/Mo/O ratio is 1 : 1.30 : 4.62, further corroborated the integrate component of Na−Mo−O. The C amount of 3.53 % should be originated from graphene oxide in the composites.

**Figure 1:**
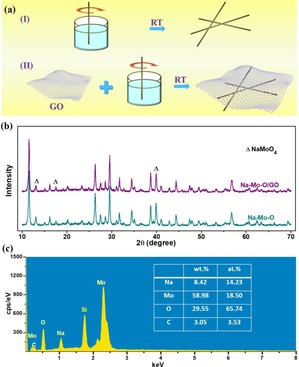
(a) Schematic illustration of simple engineering process of the Na−Mo−O nanorods and Na−Mo−O/GO composites. (b) X‐ray diffraction (XRD) patterns of the Na−Mo−O nanorods and Na−Mo−O/GO composites. (c) Energy dispersive X‐ray spectrum (EDX) of the Na−Mo−O/GO composites.

Field emission scanning electron microscopy (FESEM) and transmission electron microscopy (TEM) were conducted to learn about the morphology and detailed microstructure of the as‐prepared samples. As commonly observed, FESEM images in Figure 2a suggested the high yield (nearly∼100 %) of the nanorod morphology. The high magnification SEM images in Figure 2b and 2c reveals the nanorods are of highly uniform with an average diameter of ∼150 nm. Considering that its length could reach 100 μm, the aspect ratio of the nanorods is as high as 700, which indicates the intrinsic nature of high mechanical capacity. However, the nanorods are apt to form the bundle with the adjacent ones as shown in Figure 2c. TEM image in Figure 2d gives a clearer information that the nanorods are not well‐dispersed and aggregated into bundle instead. For comparison, in the presence of GO, the obtained Na−Mo−O nanorods shows a better dispersibility. As shown in the FESEM images of Figure 2e, 2 f and Figure S1 (*Supporting Information*), the nanorods were interconnected with the ultra‐thin GO substrate and thus show a good dispersity with each other. TEM images in Figure 2g and 2 h further revealed that the nanorod bundle disappeared and instead Na−Mo−O nanorods are well‐wrapped by GO. We believed that the modification by GO has effectively improve the dispersity of the Na−Mo−O nanorods and the electric conductivity of the composites, the formed 1D nanorods interlinked with 2D GO interlayers improves the ionic and electronic transport and prevents nanoparticles pulverization and aggregation during delithiation and lithiation, which dominant the electrochemical performance in LIB testing experiment.

**Figure 2:**
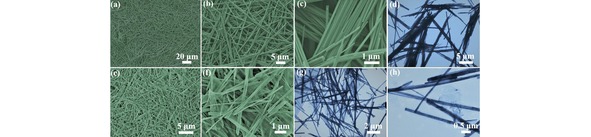
(a–c) Low and high magnification SEM and (d) TEM images of the Na−Mo−O nanoorods. (e, f) Low and high magnification SEM and (g–h) TEM images of the Na−Mo−O nanorods /GO composites.

To illuminate the enhanced electrochemical performances, the galvanostic discharge‐charge behaviors were conducted in the Li half‐cell. Figure 3a shows galvanostic discharge‐charge profiles of the Na−Mo−O/GO composites for the initial several cycles, which was carried out at a current density of 100 mA g^−1^ in the voltage window of 0.01‐3.0 V. The first discharge/charge capacity of the Na−Mo−O/GO composites is 1815/1347 mAh g^−1^ at 100 mA g^−1^, with an initial columbic efficiency of 74.2 %. The low efficiency should originate from the irreversible Li^+^ insertion reaction of Na−Mo−O^16^ and the Li^+^ consumption of solid electrode interfaces (SEI).^17^ The subsequent cycling profiles were almost superimposable, indicating the high reversibility of the Na−Mo−O/GO composites. For comparison, the electrode made of the bare Na−Mo−O nanorods only delivered the first capacity of 1634/1144 mAh g^−1^, and exhibit the irreversible capacity in the following cycles (shown in Figure 3b). The cycling performances of both the bare Na−Mo−O nanorods and Na−Mo−O/GO composites were also investigated at the current of 100 mA g^−1^ and 500 mA g^−1^. As shown in Figure 3c, after 100 cycles, the Na−Mo−O/GO composites delivers a capacity of 718 mAh g^−1^. In contrast, the bare Na−Mo−O electrodes shows a severe capacity decay, which deliver only 332/320 mAh g^−1^ after 100 cycles. Noticeably, a capacity increase appears at 20^th^ charge/discharge cycle during the cycling process, which attributes to the active process due to its low and sluggish kinetic properties of the bare Na−Mo−O nanorods. When the current density increased to 500 mA g^−1^, as shown in Figure 3d, the discharge capacity of Na−Mo−O/GO composites decreased gradually and stabilized at around ∼650 mAh g^−1^ after 50 cycles and kept stable in the subsequent cycles and retain a value as high as 570 mAh g^−1^ after 500 cycles. By comparison, the Na−Mo−O nanorods sample exhibits a high value in the initial cycles, whereas it shows a dramatic decrease thereafter. The large, irreversible capacity loss arising during the initial cycles, which is not uncommon for redox conversion type anodes,^18^, ^19^, ^20^ is likely due to the irreversible reaction during the 1^st^ cycle, the difficult dissolution of the SEI, as well as other factors, such as the intrinsic nature of the materials, kinetic limitations and cation deficient. Besides, we have also investigated the rate capability of the Na−Mo−O/GO composite. For example, the discharge capacities could reach as high as ∼1170 mAh g^−1^ and 455 mAh g^−1^ in Figure S2 in the Supporting Information when the current densities increased stepwise from 100 to 1000 mA g^−1^, respectively. Morever, it could be retained to 892 mAh g^−1^ when the current density returned back to 100 A g mA g^−1^. The superior performance of Na−Mo−O/GO composite is response to combination effect of Na−Mo−O nanorods and GO sheets, in which flexible GO could accommodate the volume change and prevent the aggregation of nanosized particles, and 1D ultralong Na−Mo−O nanorods supply large surface‐to‐volume ratio and shortened ion/ charge diffusion distance for Li^+^ upon lithiation and delithiation processes.

**Figure 3:**
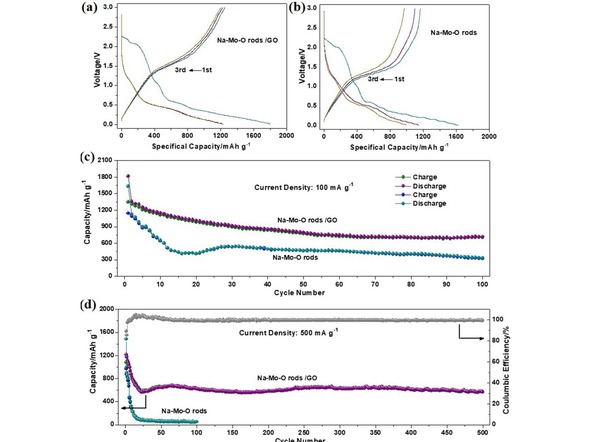
Galvanostic discharge‐charge curves of (a) Na−Mo−O/GO composites and (b) Na−Mo−O nanorods for the initial several cycles. Cyling stability of the electrodes made of bare Na−Mo−O nanorods or Na−Mo−O/GO composites at the current of (c) 100 mA g^−1^ and (d) 500 mA g^−1^.

The Nyquist plots of the Na−Mo−O/GO composites and Na−Mo−O nanorods at different stages are exhibited in the electrochemical impedance spectroscopy (EIS) in Figure 4. Incorporating with the modified Randles equivalent circuit in Figure 4a, the kinetic properties can be further studied. Before cycling as shown in Figure 4b, the Na−Mo−O/GO composites show the smaller transfer resistance than the bare Na−Mo−O nanorods, an indication of GO combinations related to lower resistance. After 50 cycles as shown in Figure 4c, the charge transfer resistance (*R*_ct_) (Figure 4c) of the Na−Mo−O/GO composites is 292 Ω, which is about 3 times smaller than the bare Na−Mo−O nanorods. Noticeably, with the cycle increasing, the Rct of both the bare Na−Mo−O nanorods and Na−Mo−O nanorods /GO composites shows the obvious increase, indicating that the serious pulverization occurs in the electrodes. The lower resistance of the Na−Mo−O/GO than the bare Na−Mo−O should be attributed to the improved electronic conductivity of the electrode due to the combination with GO. A comparison of electrochemical behaviors of the Na−Mo−O/GO composites and Na−Mo−O nanorods corroborates well with the EIS testing result, and suggest that flexible GO could not only accommodate the volume change and prevent the aggregation of nanosized particles, but also provide more active site for lithium‐ion storage.

**Figure 4:**
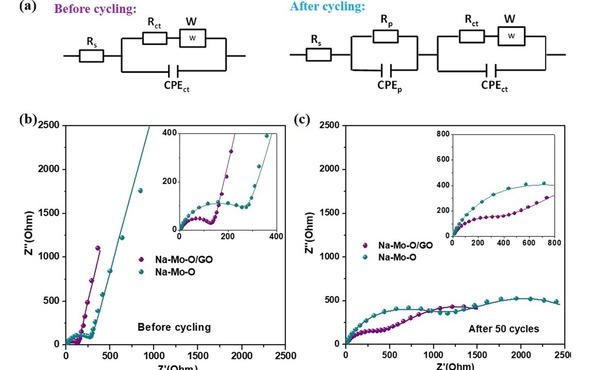
(a) The equivalent circuit model before cycling (right) and after cycling (left) (Rs: electrolyte resistance, Rp: SEI film resistance, Rct: charge transfer resistance at the surface of electroactive materials, CPEp and CPEct: capacitance, and W: Warburg impedance). Nyquist plots of AC impedance spectra of the Na−Mo−O/GO composites and the bare Na−Mo−O nanorods at different stages: before cycling (b), and after 50 cycles (c). All the samples after cycling were at the fully‐charged status.

In summary, we have synthesized the Na−Mo−O nanorods/GO composite via a simple method, which involves the in‐situ co‐precipitation and room temperature reaction. Moreover, the as‐obtained composites exhibited an obvious improvement on the electrochemical performance in comparison with the bare Na−Mo−O nanorods. On the one hand, the introduction of graphene oxide could not only increase the electronic conductivity of the electrode, but also act as the reservoir to accommodate more lithium ions. On the other hand, the synergistic effect of GO and Na−Mo−O nanorods nanoparticles also play a key role to achieve impressive lithium storage. Considering the easy‐scalability and low producing cost, the Na−Mo−O nanorods/GO composite may show great prospective in future.

## Experimental Section

All the chemical reagents were purchased from Sino Chem Cor. without any further purification.

### Materials Synthesis

Na−Mo−O nanorods were prepared by a simple precipitation reaction at room‐temperature. In a typical procedure, 0.75 g ammonium molybdate tetrahydrate ((NH_4_)_6_Mo_7_O_24_ ⋅ 4H_2_O) and 0.25 g sodium chloride (NaCl) were separately dissolved into 8 mL distilled water to form a transparent solution (denoted as solution A and solution B, respectively). Then solution B was poured into the solution A under stirring and kept at room temperature for 12 h. Finally the white precipitation was washed by water for several times and vacumm‐freezing dried for further characterization. For the Na−Mo−O nanorods/GO composites, 0.3 g graphene oxide (GO) sample prepared using a modified Hummer's method^21^ was firstly sonically dispersed into the solution A then followed the as‐aforementioned steps to obtained the Na−Mo−O nanorods/GO composites.

### Characterization

The morphology of the composites was examined by field emission scanning electron microscopy (FESEM) on a HITACHI S‐4800 SEM, by transmission electron microscopy (TEM) on a JEOL 2100F microscope. The composite crystal structures were determined by X‐ray diffraction (XRD) on a BRUKER D8 ADVANCE (Germany) instrument using Cu Ka radiation. Energy disperse X‐ray spectrum was recorded using the EDX spectrometer (Bruker AXS Flash Detector 5030) attached to the SEM.

### Electrochemical Measurements

The working electrodes were consisted of active material (Na−Mo−O nanorods or Na−Mo−O nanorods/GO, 70 wt%), conductive agent (super P, 20 wt%), and the binder (carboxyl methylated cellulose (CMC), 10 wt%). Lithium disk were used as the counter electrode, and 1 M LiPF_6_ in ethylene carbonate (EC)/diethyl carbonated (DEC) (1 : 1, v/v) as the electrolyte. The coin cells (CR2025) were aged for 10 h before measurement. Impedance spectroscopy (EIS) was carried out using Ivium Vertex C electrochemical workstation (Ivium, Netherlands). The galvanostatic charge/discharge profiles were performed on a Neware testing system (BTS‐4008).

## Conflict of interest

The authors declare no conflict of interest.

## Supporting information

As a service to our authors and readers, this journal provides supporting information supplied by the authors. Such materials are peer reviewed and may be re‐organized for online delivery, but are not copy‐edited or typeset. Technical support issues arising from supporting information (other than missing files) should be addressed to the authors.

Supplementary
