# Supplementary material for: Engineering Na−Mo−O/Graphene Oxide Composites with Enhanced Electrochemical Performance for Lithium Ion Batteries
Source: ChemistryOpen. 2019 Aug 29;8(10):1225–9. doi: 10.1002/open.201900205 (PMC6769431; doi:10.1002/open.201900205)
Supplement: Supplementary file 1 — Supplementary [file OPEN-8-1225-s001.pdf]

ChemistryOPEN
Open Access

Supporting Information

© Copyright Wiley-VCH Verlag GmbH & Co. KGaA, 69451 Weinheim, 2019

Engineering Na–Mo–O/Graphene Oxide Composites with Enhanced Electrochemical Performance for Lithium Ion Batteries

Jingfa Li,* Qiang Chen, Qihao Zhou, Nan Shen, Min Li, Cong Guo, and Lei Zhang©2019 The Authors. Published by Wiley-VCH Verlag GmbH & Co. KGaA.
This is an open access article under the terms of the Creative Commons Attribution License, which permits use, distribution and reproduction in any medium, provided the original work is properly cited.

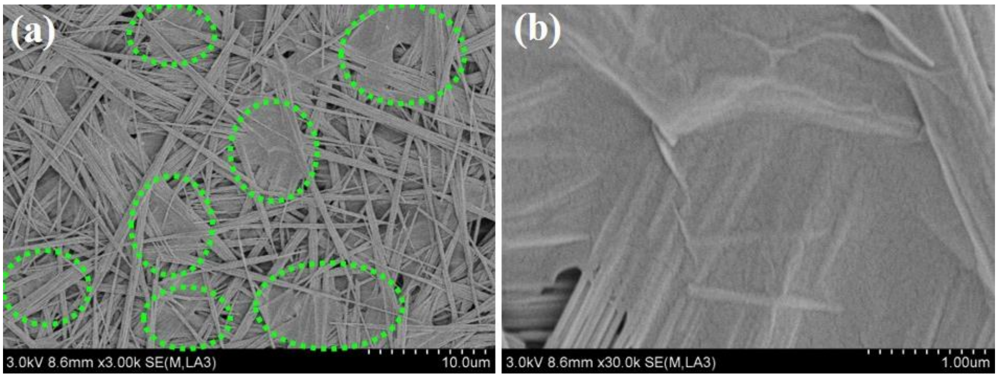

Figure S1. Additional low and high magnification SEM images of the Na-Mo-O nanorods /GO composites.

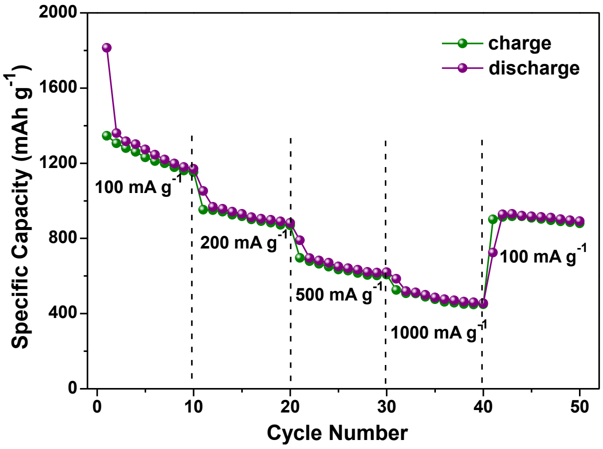

Figure S2. Rate performance of the electrode made of Na-Mo-O nanorods /GO composites.
